# Two Best Doctors

**Published:** 1860-07

**Authors:** 


### THE TWO BEST DOCTORS.

For all minor aches and ails, Dr. Letalone is the most uniformly and happily successful physician I ever knew; but in the severer forms of disease it is always wisest, safest and best to seek promptly the advice of an educated practitioner; and a fortunate thing would it be for humanity, if not an atom or a drop of physic were ever taken, unless specially prescribed by those who had the advantage of a thorough medical education.

